# Expression of Oxytocin/Neurophysin I and Oxytocinase in the Equine Conceptus from Day 8 to Day 21 Post-Ovulation

**DOI:** 10.3390/ani12070799

**Published:** 2022-03-22

**Authors:** Mariana Diel de Amorim, Claudia Klein, Robert Foster, Lynn Dong, Maria Fernanda Lopez-Rodriguez, Claire Card

**Affiliations:** 1 Department of Clinical Sciences, Cornell University, 930 Campus Rd, Ithaca, NY 14853, USA; 2 Department of Veterinary Clinical and Diagnostic Science, University of Calgary, 2500 University Drive NW, Calgary, AB T2N 1N4, Canada; claudia.klein@fli.de; 3 Department of Pathobiology, Ontario Veterinary College, University of Guelph, 50 Stone Road E, Guelph, ON N1G 2W1, Canada; rfoster@uoguelph.ca; 4 Immunopathology Research and Development Laboratory, Department of Biomedical Sciences, Cornell University, 930 Campus Rd, Ithaca, NY 14853, USA; lynn.dong19@gmail.com; 5 Department of Large Animal Clinical Sciences, Western College of Veterinary Medicine, 52 Campus Drive, Saskatoon, SK S7N 5B4, Canada; maria.lopez@usask.ca

**Keywords:** equine conceptus, glucose transporters, leucyl and cystinyl aminopeptidase, oxytocin/neurophysin I, prostaglandin

## Abstract

**Simple Summary:**

Oxytocinase is an enzyme that breaks down oxytocin in serum and tissues. The presence of oxytocin and oxytocinase mRNA and protein and their relationship to other genes in the horse embryo/conceptus that regulate pregnancy recognition and embryo/conceptus fixation have not been investigated. Our objective was to characterize the expression of oxytocin, oxytocinase and other related genes in horse embryos. Oxytocinase was increased at D15 compared to D10 and was identified in the horse embryo membranes. Oxytocin expression was highest at D21, and lowest on D12 and D14; no protein was identified, while its receptor (OXTR) was highest on D14 and D21. Oxytocinase was highest at D15 compared to D10 and high expression of prostaglandin E synthase was found on D14, D15 and D21. Oxytocinase may have a role in embryonic fixation. Further investigation of the role of oxytocinase during pregnancy is warranted.

**Abstract:**

Leucyl and cystinyl aminopeptidase (LNPEP/oxytocinase) is an enzyme that metabolizes oxytocin in serum and tissues. The presence of oxytocin/neurophysin I (OXT), oxytocin and LNPEP and their relationship to other genes is unknown in the equine conceptus. Our objective was to characterize gene expression of LNPEP and OXT on D8, 10, 12, 14, 15, 16 and 21 conceptuses in relationship to other genes. Immunohistochemistry, western blot and liquid chromatography with tandem mass spectrometry (LC-MS/MS) were used for identification of oxytocin and LNPEP in D15, 16 and 18 conceptuses. LNPEP was increased at D15 compared to D10, was immunolocalized in the equine trophectoderm and endoderm, and protein was confirmed by LC-MS/MS. Maximal abundance of OXT was at D21, and lowest on D12 and D14, but no protein was identified. OXTR abundance was highest on D14 and D21. LNPEP was correlated with PTGFR and PTGES on D12 and D14–D15, and high expression of PTGES, PTGS2 was found on D14, D15 and D21; PTGFR was found on D8 and D12–21. LNPEP may have a role in prostaglandin regulation and conceptus fixation by decreasing the availability of oxytocin. Further investigation on the role embryonic LNPEP during pregnancy is warranted.

## 1. Introduction

Maternal recognition of pregnancy (MRP) has been investigated in the mare, but unlike other domestic animal species, the precise conceptus-driven pathway responsible for blocking the luteolytic mechanism is unknown. The inability to block luteolysis invariably leads to early embryonic death. Embryonic loss has negative, costly economic impacts on the equine industry [1]. Understanding the physiology of MRP in mares may provide information that will lead to the development of future therapies that will ameliorate such losses. Investigators have characterized the presence, expression or secretion of steroid hormones [2,3], steroid hormone receptors [4], proteins [5,6], prostaglandins [7,8,9] and oxytocin [10] in the early equine conceptus. The early conceptus secretes estrogen as early as Day 6 [3], and estradiol (E2), estrone (E1) and their sulfates (E_2_S and E_1_S) are secreted on D11–D26 [2]. The embryo proper is capable of metabolizing estrogen that is present in the yolk sac [2]. Estradiol was investigated as a MRP factor; however, administration of estradiol 17-β into the uterine lumen of the mare failed to extend luteal function [11,12]. The equine trophectoderm reportedly expresses *ESR2* (ERβ) on Day 14–16 but not *ESR1* (ERα), while the progesterone receptor (*PGR*) was expressed in all stages of the conceptus examined [4].

Uterine tubal transport of the equine embryo is initiated by embryonic prostaglandin E2 (PGE) secretion [13]. The developing equine conceptus continues to secrete high concentrations of PGE2 in the uterus [7,9,14]. Prostaglandin E2 was investigated as a MRP factor, however intrauterine administration of PGE2 to diestrus mares failed to prolong luteal function [11,15]. Prostaglandin E2 and/or F2 (PGF2α) produced by the conceptus are associated with uterine propulsion of the embryo [14,15,16]. Non-selective inhibitors of prostaglandin synthesis reduce embryo mobility [17]. Uterine transport of D13 embryos was greater than simulated sham vesicles and simulated vesicles releasing PGE [11]. The mechanism responsible for fixation of the equine conceptus is unknown, but the secretion of the hormones implicated in uterine transport, PGE2 and PGF2α, continues past the time of conceptus fixation [14], indicating that there is another regulatory step responsible for the cessation of mobility and embryonic fixation. Further studies are needed to understand conceptus fixation and events during early pregnancy.

In mares, there is a positive feedback loop during luteolysis and parturition involving oxytocin, OXTR and endometrial PGF2α secretion [18,19,20]. De Ruijter-Villani and co-authors (2015) reported in D14 pregnant mare endometria that downregulation of the PGF receptor intercepted the oxytocin-prostaglandin F feedback loop [21]. Through proteomics, prostaglandin F2 receptor inhibitors (PTGFRN) and a progesterone potentiating protein (FK506) were identified in the equine blastocoel fluid of the D13 conceptus [6], along with growth factors and cytokines in blastocoele and yolk sac fluid [5]. The decrease in endometrial *PTGFR* and presence of conceptus PTGFRN would be expected to decrease the function of PGF2α due to the lower number of receptors and presence of PTGFR inhibitors.

Oxytocin has important and diverse physiologic roles in many species. Oxytocin is secreted by the neurohypophysis in mammalian species and oxytocin/neurophysin I is found in luteal tissue in ruminants [22,23]. Pituitary secretion of oxytocin is associated with large peaks of endometrial (PGF2α) [24]. Circulating free oxytocin binds to oxytocin receptors and stimulates myometrial contractility [25]. More recently, an in vitro study of primary trophoblast cell cultures showed that oxytocin had no major effect on the expression of enzymes controlling prostaglandin synthesis [7]. The local release of conceptus or endometrial oxytocin may have a role, however, in promoting endometrial or myometrial prostaglandin release [18], and may also affect the transport of the equine conceptus. The role of oxytocin in equine pregnancy and in the luteolytic mechanism is not fully known [1,26]. Exogenous oxytocin administered during the midluteal phase prolongs luteal function in mares [27,28,29]. Oxytocin has been identified in equine blastocoel fluid [30] and uterine flush fluid [31] by radioimmunoassay. There are conflicting reports in mares regarding the presence of luteal and endometrial oxytocin and its carrier protein neurophysin I [32,33]. However, in ruminants, oxytocin/neurophysin I is not expressed in conceptus tissues [34], but is present in characteristic granules in luteal tissue [22,23]. The secretion of luteolytic prostaglandin in nonpregnant mares is believed to be mediated through a long progesterone exposure followed by the coupling of oxytocin with its receptor (OXTR) in the endometrium [18]. The expression of the receptor for oxytocin (*OXTR*) has been demonstrated in the equine conceptus trophoblast by Budik and co-authors (2012) [26].

There is little information on the key enzymes that regulate local and systemic oxytocin concentrations in horses. Leucyl and cystinyl aminopeptidase (LNPEP), which is also called oxytocinase, metabolizes oxytocin. LNPEP is both a soluble and transmembrane aminopeptidase that cleaves and inactivates many molecules, including oxytocin [35]. The LNPEP is produced by the endoplasmic reticulum and has been identified in glucose transporter 4 (GLUT4) vesicles. Transport of LNPEP in GLUT4 vesicles occurs in a process that is stimulated by insulin and results in exocytosis of LNPEP in some species [36,37]. Expression of glucose transporters GLUT1 and GLUT4, also known as *SLC2A1* and *SLC2A4*, respectively, has been identified in the ovine endometrium. The secretion of GLUT4 vesicles is dependent on progesterone, along with the conceptus factor IFNτ and prostaglandins [38,39]. A recent study demonstrated that the equine conceptus expresses glucose transporters on Days 7–28 [40]. However, no studies have investigated whether *LNPEP* is present in the GLUT4 vesicles in the equine conceptus. There have been no studies evaluating changes in *LNPEP* and LNPEP in the equine embryo/conceptus in early pregnancy and at the time of conceptus fixation.

We hypothesized that *LNPEP* would be expressed at higher levels by the conceptus at the expected time of conceptus fixation, and the gene *OXT* would be expressed in the equine conceptus. Our objectives were to characterize changes in and examine relationships between *LNPEP* and *OXT* and their protein products during early pregnancy. A second objective was to examine relationships with *LNPEP* and *OXT* and the expression of important putative regulators in pregnancy: steroid hormone receptors, prostaglandin related genes and glucose transporters in the equine embryo/conceptus from Day 8 through 21 post-ovulation.

## 2. Materials and Methods

### 2.1. Animals

Animals were housed in two locations. The main group of mares (n = 21) was housed at the Goodale Research Farm, Saskatoon, SK, Canada (latitude N 52°2′ and longitude W 106°42′) for mRNA and protein studies. Mares were housed together and lived outside with assess to sheds for shelter and were fed free choice hay, a small amount of grain, water and trace mineral supplementation to meet their nutritional requirements. Housing and handling of all animals were consistent throughout all the experimental periods. Mares were handled and trained daily so they became accustomed to the chute and stocks systems of the research farm in the two months prior to the start of experimental period. These mares ranged in age from 2 to 19 years, with a mean and standard deviation (±SD) age of 10.1 (±4.3) years. Mares were light horse breeds, with the majority being Quarter Horses, and additionally there was one Appendix Quarter Horse, one Thoroughbred and one Warmblood mare.

A second group of Standardbred and Thoroughbred mares (n = 9) were maintained on pasture from May to November at the University of Guelph, Guelph, ON, Canada (latitude N 43°31′ and longitude W 80°13′) for the purpose of conceptus collection for IHC.

During the physiological breeding season of the Northern hemisphere (from May until November), reproductive ultrasound examinations were performed every other day until the mares were determined to be in estrus (follicle ≥30 mm with at least a grade 1 uterine edema, soft uterus with no corpus luteum present). During estrus, daily reproductive examination was performed. When a 35 mm follicle with grade 2 edema was identified, mares received a single injection of an ovulatory induction agent (deslorelin, 1.5 mg, intramuscularly) and were bred with semen from a single fertile stallion (>250 million progressively motile morphologically normal sperm) every other day until the day of ovulation (D0). Mares were randomly assigned to groups for an embryo/conceptus collection on day(D): D8 (n = 4), D10 (n = 5), D12 (n = 5), D14 (n = 6), D15 (n = 4), D16 (n = 7), and D21 (n = 3) for embryonic/conceptus gene expression studies. Additionally, nine conceptuses (n = 3 per sample day) were collected on D15, 16 and 18 post-ovulation for protein analysis. The second group of mares were bred at the University of Guelph for the purpose of conceptus collection on D15, D16 and D18.

### 2.2. Transcervical Embryo/Conceptus Collection

Conceptus diameter was measured daily with a transrectal ultrasound in a 90° angle, and a mean of three measurements was calculated through the day of collection except for D8 embryos, which were too small to be detected by ultrasound. Mares determined to be pregnant by identification of a conceptus were placed in the stocks and their tails were wrapped. The perineum was thoroughly cleansed. An equine uterine bivona catheter (Bivona, Partnar Animal Health, Ilderton, ON, Canada) was placed per vaginum through the cervix and the cuff was inflated with sterile water to seal the cervix. The bivona catheter was connected by a long Y-tubing (Long Foley Y-Junction with Tubing, Partnar Animal Health, Ilderton, ON, Canada) to 1 L bags of lactated ringer’s solution. A liter of lactated ringer’s solution was gravity fed into the uterus through the catheter, and then the fluid was recovered and passed through a 75 μm filter (EmCon Filter, Partnar Animal Health, Ilderton, ON, Canada). Embryo/conceptuses were removed from the filter and placed in a petri dish. For D8 embryos, a stereomicroscope with a 10–50× magnification was used. All embryos/conceptuses were rinsed with PBS several times, and D8 embryos were placed in a cryovial and snap frozen in liquid nitrogen whole. The other conceptuses had their capsule removed and were frozen separately. Conceptuses were then stored at −80 °C until analysis. Other equine conceptuses (n = 9) of varying ages (D15, D16 and D18) were also collected in the same fashion for protein analysis through western blot (WB), immunohistochemistry (IHC) and liquid chromatography tandem mass spectrometry (LC-MS/MS). Conceptuses for the WB and LC-MS/MS experiments were snap frozen and stored at −80 °C until analysis. Conceptuses (n = 9) for IHC were collected as above using PBS, placed in 10% formol buffered saline for 24 h, and then embedded in paraffin.

### 2.3. RNA Isolation

Total RNA was extracted using AllPrep^®^ (Qiagen, Toronto, ON, Canada) reagent (TissueLyser LT, Qiagen, Toronto, ON, Canada) according to the manufacture’s procedural guidelines. An equal volume of 70% ethanol was mixed with the homogenate and transferred to an RNeasy spin column for three-step buffer add-on solution and centrifugation according to manufacturer’s protocol. RNase-free water was added to the spin column as the final step to elute the RNA. Concentration of the RNA was assessed via NanoDrop (Thermo Fisher Scientific, Ottawa, ON, Canada). Samples with 260/280 and 260/230 ratios between 1.8–2.0 and 1.9–2.2, respectively, were considered pure and used for the analyses.

### 2.4. Real-Time RT-PCR (qPCR)

Briefly, 200 ng of RNA sample was incubated with DNase I, Amplification Grade for 15 min at room temperature, according to the manufacture’s protocol. This was followed by the addition of 1 μL of 25 mM EDTA solution to and heating of the solution for 10 min at 65 °C. The prepared sample was used for reverse transcription using a High-Capacity cDNA Reverse Transcription Kit (Thermo Fisher Scientific, Ottawa, ON, Canada), according to the manufacture’s user’s guide. All PCR reactions were performed in duplicate, and no template and no-RT controls were included.

The mRNA expression was measured by real-time PCR of the following transcripts: estrogen receptor 1 (*ESR1*), progesterone receptor (*PGR*), oxytocin receptor (*OXTR*), oxytocin/neurophysin I prepropeptide (*OXT*), leucyl and cystinyl aminopeptidase (*LNPEP*), prostaglandin-endoperoxide synthase 2 (*PTGS2*), prostaglandin F receptor (*PTGFR*) and prostaglandin E synthase (*PTGES*), solute carrier family 2 member 4 (*SLC2A4*) and solute carrier organic family member 2A1 (*SLC2A1*). The sequence of the *PGR, OXTR, PTGES* and *PTGS2* primers were used from previously published papers [21,41]. All other primers for the selected transcripts above were designed with Primer-BLAST (National Center for Biotechnology Information) and are displayed in Table 1.

For quantification of the relative expression of each gene, real-time PCR was then performed using PowerUp SYBR Green Master Mix (Thermo Fisher Scientific, Ottawa, ON, Canada), according to the manufacturer’s instructions and as previously published [42]. Briefly, the reaction was prepared by mixing: 0.5 μL of cDNA (total volume of 10 μL per well), 5 μL of PowerUp™ SYBR™ Green Master Mix, 0.2 μL of 10 μM forward and reverse primers and 3.5 μL of ddH_2_O with the thermal cycling condition as follows: one cycle for 10 min at 95 °C, 40 cycles of 15 sec at 95 °C, 1 min at 60 °C, and generation of a melt curve. An automatic pipetting system was used (epMotion Automated Pipetting System, Eppendorf, Mississauga, ON, Canada) for all the reactions. A 10-fold dilution of pooled cDNA was used to determine primer PCR efficiency, which was defined as 90 to 100% PCR efficiency. Specificity of primers was evaluated by a single melting temperature, and also by performing a Sanger sequencing of each product to confirm the nucleotide sequence. The cycle number where the fluorescent signal intersected the threshold (Ct) was determined for each reaction and values from replicates were averaged. The averaged Ct of the real-time PCR was normalized to the housekeeping gene (*GAPDH*) by subtracting the Ct for *GAPDH* to obtain the delta Ct.

### 2.5. Western Blot (WB) for OXT and LNPEP

#### 2.5.1. Sample Preparation

Combined equine embryos/conceptuses of different ages were homogenized using a pre-chilled mortar and pestle method by adding RIPA lysis buffer supplemented with a mixer of protease inhibitors (Santa Cruz, Dallas, TX, USA) at a ratio of 1 g of tissue to 3 mL of lysis buffer. The homogenized embryonic/conceptus tissues were transferred to small tubes, incubated on ice for 30 min and vortexed every 10 min. The total crude protein supernatants were obtained after centrifugation at 15,000 rpm for 10 min. The total crude protein extracts were aliquoted and stored at −20 °C until analysis.

#### 2.5.2. Determination of Protein Concentration

The total protein concentrations from crude extracts were determined with Pierce Detergent Compatible Bradford Assay Kit (Thermo Scientific, Delaware, MA, USA) based on a standard curve obtained from a series of dilutions of bovine serum albumin (BSA) by following the manufacturer’s instructions.

#### 2.5.3. Gel Electrophoresis and Protein Transfer

The crude protein extracts (20 μg) were denatured in sample loading buffer and separated using electrophoresis in a polyacrylamide gel system (4–20% Mini-PROTEAN^®^ TGXTM Precast Gels with Mini-PROTEAN^®^ Tetra Cell using Tris/Glycine/SDS Electrophoresis Buffer (Bio-Rad, Hercules, CA, USA). The separated proteins were transferred onto Immobilon-P polyvinylidene difluoride (PVDF) membranes (Millipore, Bedford, MA, USA) in Tris/glycine buffer with Mini Trans-Blot^®^ Cell (Bio-Rad, Hercules, CA, USA).

#### 2.5.4. Blocking and Antibodies

The PVDF membranes containing transferred proteins were washed in phosphate buffered saline with 0.05% Tween 20 and incubated in blocking buffer (10% goat serum, 2× casein in PBS) for 1 h at room temperature to prevent nonspecific binding. Primary antibodies, rabbit anti-oxytocin (ImmunoStar, Hudson, WI, USA, 1:1000), and rabbit anti-LNPEP (LifeSpan BioSciences, Seattle, WA, USA, 1:1000) were diluted in PBS containing 1× casein and incubated overnight at 4 °C. To determine epitope-specific and non-specific binding, a PVDF membrane was treated with primary antibody and a second PVDF membrane served as the isotype negative control which was treated with normal rabbit serum diluted to an equivalent final concentration (rabbit IgG at 1:5000 for LNPEP, rabbit IgG at 1:1000 for OXT). After that, the membranes were washed three times in PBS containing 0.05% tween 20 for 5 min each and incubated with biotinylated goat at rabbit IgG (Vector Laboratories, Burlingame, CA, USA, 1:1000 in PBS) for 30 min, followed by incubation with streptavidin conjugated to alkaline phosphatase (Vector Laboratories, Burlingame, CA, USA, 1:500 in PBS) for 20 min at room temperature. Positive staining was visualized by incubation with ImmPACT^®^ Vector^®^ Red chromogen (Vector Laboratories, Burlingame, CA, USA). Precision Plus ProteinTM Dual Color Standards (Bio-Rad, Hercules, CA, USA) were used for monitoring gel electrophoresis and confirmation of proper protein transfer. A separate blot against mouse anti-GAPDH (Santa Cruz, Santa Cruz, Dallas, TX, USA, 1:1000) was used as a loading control.

### 2.6. Liquid Chromatography Tandem Mass-Spectrometry (LC-MS/MS)

A Coomassie blue-stained polyacrylamide gel was run side by side with the polyacrylamide gel used for WB to identify immune-positive bands against the antibodies used. Each corresponding band in the polyacrylamide gel of the expected molecular weight (MW) for OXT (MW of 10–17 kDa) and LNPEP (MW of 115–150 kDa) were excised and submitted for liquid chromatography tandem mass spectrometry (LC-MS/MS) for protein identification to the Proteomics Facility at the Institute of Biotechnology at Cornell University.

#### 2.6.1. In-Gel Trypsin Digestion of SDS Gel Bands

The protein bands (~1 mm cubes) from an SDS-PAGE gel were subjected to in-gel digestion followed by extraction of the tryptic peptide, as reported previously [43]. The excised gel pieces were washed consecutively with 200–400 μL distilled/deionized water, followed by 50 mM ammonium bicarbonate, 50% acetonitrile and finally 100% acetonitrile. The dehydrated gel pieces were reduced with 50–400 μL of 10 mM DTT in 100 mM ammonium bicarbonate for 1 h at 56 °C, and alkylated with 50–400 μL of 55 mM iodoacetamide in 100 mM ammonium bicarbonate at room temperature in the dark for 45 min. After repeated wash steps as described above, the gel slices were then dried and rehydrated with trypsin (Promega), at an estimated 1:3 w/w ratio in 50 mM ammonium bicarbonate, 10% ACN and incubated at 37 °C for 18 hrs. The digested peptides were extracted twice with 200 μL of 50% acetonitrile, 5% formic acid and once with 200 μL of 75% acetonitrile, 5% formic acid. Extractions from each sample were combined and filtered with 0.22 um spin filter (Costar Spin-X from Corning) and dried in the speed vacuum. Each sample was reconstituted in 2% acetonitrile, 0.5% formic acid prior to LC-MS/MS analysis.

#### 2.6.2. Protein Identification by Nano LC/MS/MS Analysis

The in-gel tryptic digests were reconstituted in 20 μL of 0.5% FA for nano LC-ESI-MS/MS analysis, which was carried out using an Orbitrap FusionTM TribridTM (Thermo-Fisher Scientific, San Jose, CA, USA) mass spectrometer equipped with a nanospray Flex Ion Source, and coupled with a Dionex UltiMate3000RSLCnano system (Thermo, Sunnyvale, CA, USA) [44,45]. The gel-extracted peptide samples (5 μL) were injected onto a PepMap C-18 RP nano trapping column (5 μm, 100 μm i.d × 20 mm) at 15 μL/min flow rate for rapid sample loading and then separated on a PepMap C-18 RP nano column (2 μm, 75 μm × 25 cm) at 35 °C. The tryptic peptides were eluted in a 60 min gradient of 5% to 38% acetonitrile (can) in 0.1% formic acid at 300 nL/min., followed by a 7 min ramping to 90% ACN-0.1% FA and an 8 min hold at 90% ACN-0.1% FA. The column was re-equilibrated with 0.1% FA for 25 min prior to the next run. The Orbitrap Fusion is operated in positive ion mode with spray voltage set at 1.6 kV and source temperature at 275 °C. External calibration for FT, IT and quadrupole mass analyzers was performed. In data-dependent acquisition (DDA) analysis, the instrument was operated using FT mass analyzer in MS scan to select precursor ions, followed by 3 s “Top Speed” data-dependent CID ion trap MS/MS scans at 1.6 *m*/*z* quadrupole isolation for precursor peptides with multiple charged ions above a threshold ion count of 10,000 and normalized collision energy of 30%. The MS survey scans were performed at a resolving power of 120,000 (fwhm at *m*/*z* 200), for the mass range of *m*/*z* 375–1575. Dynamic exclusion parameters were set at 40 s of exclusion duration with ±10 ppm exclusion mass width. All data were acquired using Xcalibur 3.0 operation software (Thermo-Fisher Scientific).

#### 2.6.3. LC-MS/MS Data Analysis

The DDA raw files for CID MS/MS were subjected to database searches using Proteome Discoverer (PD) 2.3 software (Thermo Fisher Scientific, Bremen, Germany) with the Sequest HT algorithm processing workflow for precursor-based quantification. The PD 2.3 processing workflow containing an additional node of Minora Feature Detector for precursor ion-based quantification was used for protein identification. The database search was conducted against *Equus caballus* NCBI Jun 2018 database, having 245,782 entries. Two-missed trypsin cleavage sites were allowed. The peptide precursor tolerance was set to 10 ppm and fragment ion tolerance was set to 0.6 Da. Variable modification of methionine oxidation, deamidation of asparagines/glutamine and fixed modification of cysteine carbamidomethylation were set for the database search. Only high-confidence peptides defined by Sequest HT with a 1% FDR by Percolator were considered for the peptide identification. The final protein IDs contained protein groups that were filtered with at least two peptides per protein.

The precursor abundance intensity for each peptide identified by MS/MS in each sample were automatically determined, and their unique peptides for each protein in each sample were summed and used for calculating the protein abundance by PD 2.3 software without normalization.

### 2.7. Immunohistochemistry (IHC) for OXT and LNPEP

The immunohistochemistry of the equine conceptus was performed by the Immunopathology Research and Development Core Laboratory at Cornell University. Briefly, 4 μm thick sections of formalin-fixed/paraffin-embedded sections were used for immunohistochemical analysis. After deparaffinization in xylene and rehydration in graded ethanol, the sections were subjected to antigen retrieval by steaming in citrate buffer (10 mM, pH 6.0) for 20 min followed by 30 min cooling at room temperature. Then, endogenous peroxidase activity was quenched by 0.3% hydrogen peroxide in distilled water for 10 min. IHC analysis was performed using ImmPRESS^®^ HRP Goat Anti-Rabbit IgG (Peroxidase) Polymer Detection Kit (Vector Laboratories) following the kit instruction. The tissue sections were incubated with primary antibodies for 1.5 h at room temp on an orbital shaker. For LNPEP, the rabbit polyclonal anti-human LNPEP IgG was purchased from LifeSpan BioSciences, Inc (LSBio, Cat# LS-B12918/65145) and used at 1:400 for all conceptuses. For oxytocin, rabbit anti-synthetic oxytocin whole serum was purchased from ImmunoStar (cat#20068) and used at 1:500. Negative controls were run in parallel by replacing the primary antibody with normal rabbit IgG for LNPEP and oxytocin at the equivalent final concentration. Nova Red (Vector Laboratories) was used as chromogen to visualize antigen localization, and the sections were lightly counterstained with hematoxylin. The positive tissue control for immunostaining to detect oxytocin was the posterior pituitary gland, and for LNPEP was the anterior and posterior pituitary. All stained IHC slides were digitized with Aperio CS2 (Leica Biosystems, Buffalo Grove, IL, USA).

### 2.8. Statistical Analysis

Data analysis was performed with JMP Pro 13 (SAS Institute Inc., Cary, NC, USA). Histogram and Shapiro–Wilk tests from the residuals were analyzed for normality; the conceptus ultrasound measurements and *SLC2A1* expression data were not normally distributed, so results were reported from statistics analysis on these variables using rank transformed data. One-Way ANOVA with a post hoc Tukey test was used to compare the differences of the conceptus ultrasound measurements and the delta Ct between different Days (8–21). Multivariate Analysis (Pearson correlation) was performed to look at correlations with multiple comparisons among genes in the different gestational ages. Level of significance was set at *p* < 0.05 for all tests.

## 3. Results

**Figure 1:**
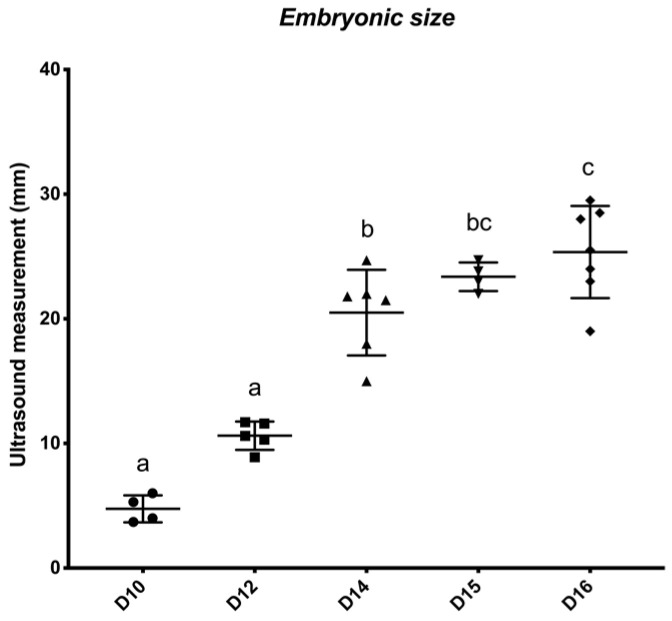
Equine embryonic ultrasound measurements (mm) by embryonic age (Day 10 to 16 post-ovulation). Differences between days (*p* < 0.05) are indicated by different superscripted letters.

A total of 34 embryos/conceptuses were collected, which included D8 (n = 4), D10 (n = 5), D12 (n = 5), D14 (n = 6), D15 (n = 4), D16 (n = 7), and D21 (n = 3). Conceptus size was statistically different among Days (*p* < 0.0001). Figure 1 demonstrates the differences between the conceptus sizes per day. Furthermore, the comparison of the embryonic/conceptus gene expression by Day is reported in Table 2.

### 3.1. Embryonic Gene Expression

#### 3.1.1. Oxytocin Related Genes

**Figure 2:**
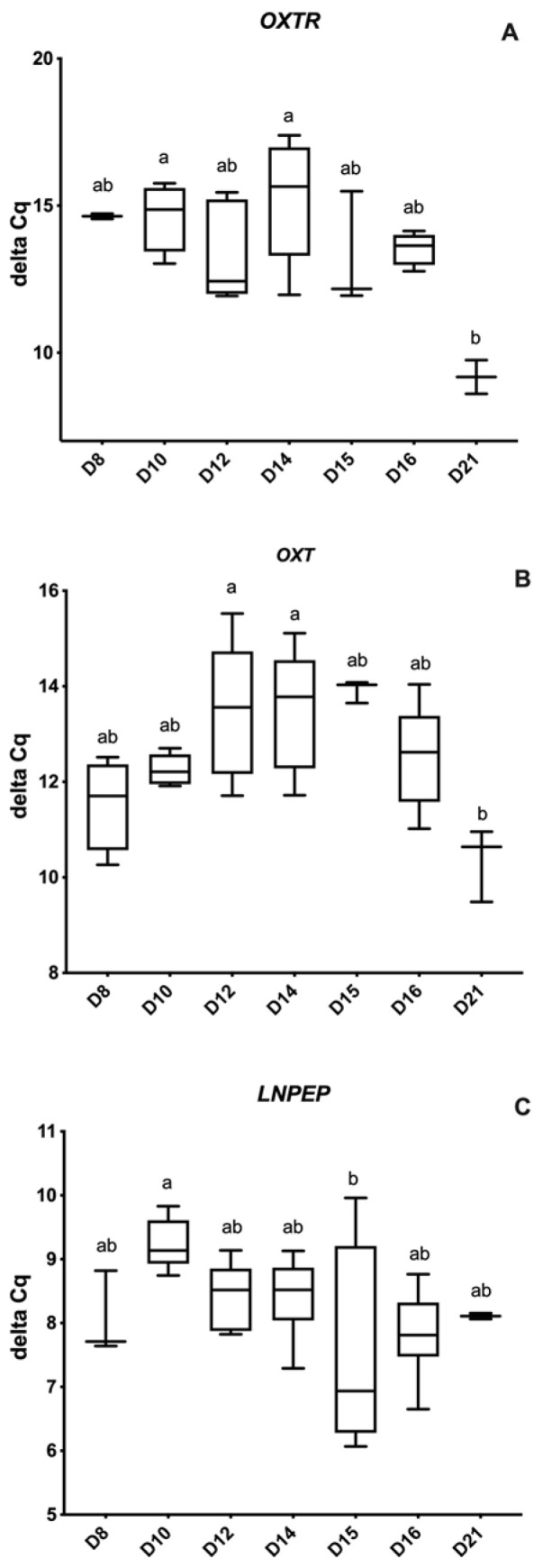
Relative abundance (delta Ct) of oxytocin-related genes by embryonic age (Days 8 to 21 post-ovulation); (**A**) Oxytocin receptor (*OXTR*), (**B**) Oxytocin/neurophysin 1 (*OXT*), and (**C**) Leucine-Cystinyl aminopeptidase (*LNPEP*). Delta Ct concentration has an inverse relationship to mRNA abundance. Differences between days (*p* < 0.05) are indicated by different superscripted letters.

Leucyl and cystinyl aminopeptidase mRNA was identified in all embryos. Regarding *LNPEP*, a lower relative abundance was noted in conceptuses on D10 compared to D15 (*p* < 0.04). Conceptuses from D10 and D14 post-ovulation had significantly lower abundance of *OXTR* compared to D21 (*p* = 0.02), and *OXT* followed a similar pattern and had decreased relative abundance in D12 and D14 conceptuses compared to D21 (*p* < 0.001). Figure 2A–C demonstrates the relative expression of *OXT*, *OXTR* and *LNPEP* among different embryonic/conceptus stages.

#### 3.1.2. Steroid Hormone Receptor

**Figure 3:**
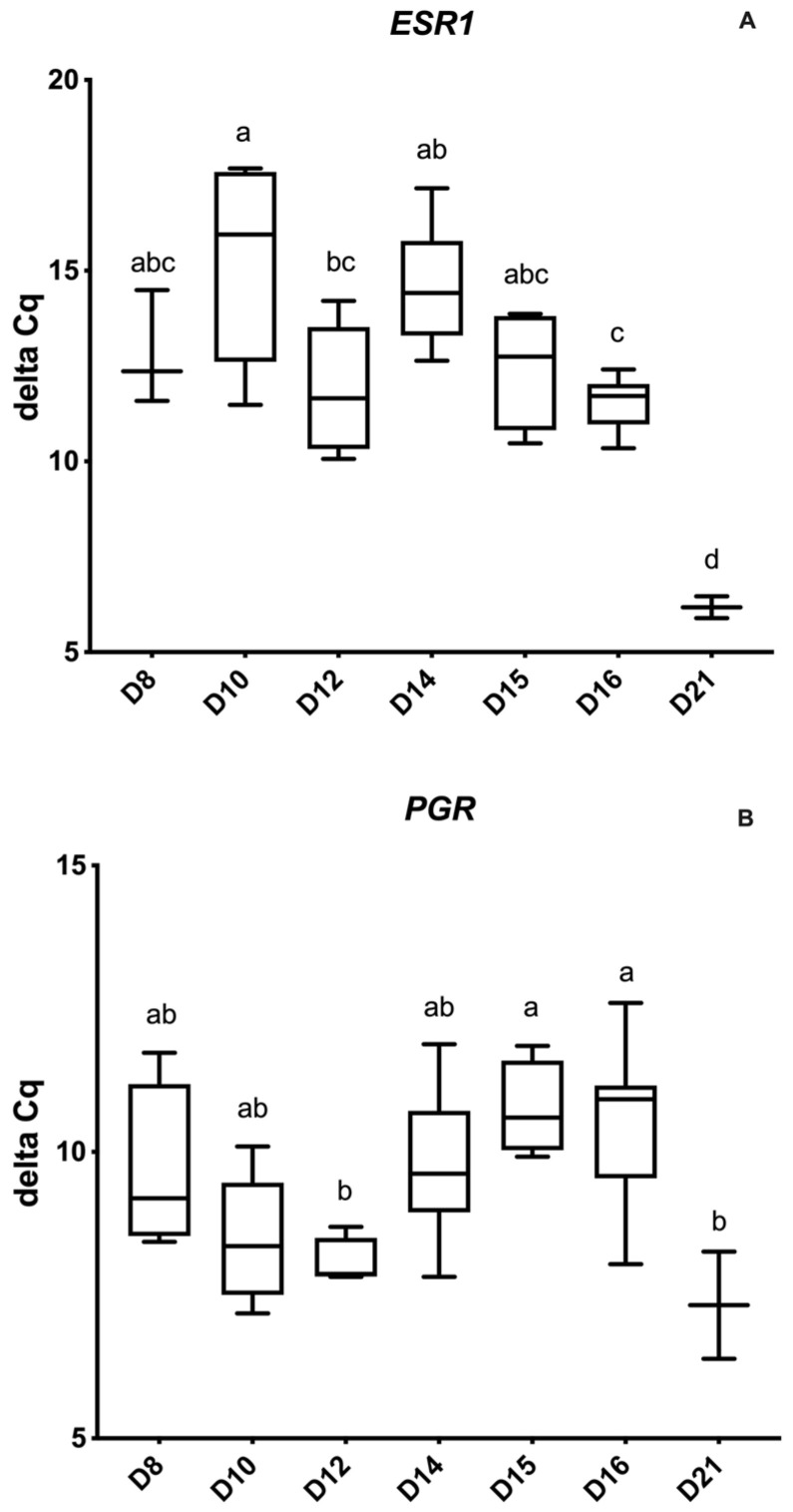
Relative abundance (delta Ct) of steroid hormone receptor genes by embryonic age (Days 8 to 21 post-ovulation); (**A**) Estrogen receptor 1 (*ESR1*) and (**B**) Progesterone receptor (*PGR*). Delta Ct concentrations have an inverse relationship to mRNA abundance. Differences between days (*p* < 0.05) are indicated by the different superscripted letters.

Figure 3 demonstrates the abundance of *ESR1* and *PGR* for the different embryonic/conceptus stages. In brief, all conceptuses expressed *ESR1* and *PGR*, and D21 conceptuses had a higher relative expression of *ESR1* compared to any other embryo/conceptus stage (*p* = 0.005). There was decreased expression of *ESR1* on D10 and D14, and an increase in the relative expression on D15. *ESR1* was significantly lower on D10 when compared to D12 and D16 (*p* = 0.03). *ESR1* was significantly increased on D12 compared to D10 and D21 (*p* = 0.04). Regarding *PGR*, there was an increase on D12 compared to D15 and D16 (*p* = 0.04), and a further increase in D21 conceptuses (*p* = 0.04).

#### 3.1.3. Prostaglandin Related Genes

**Figure 4:**
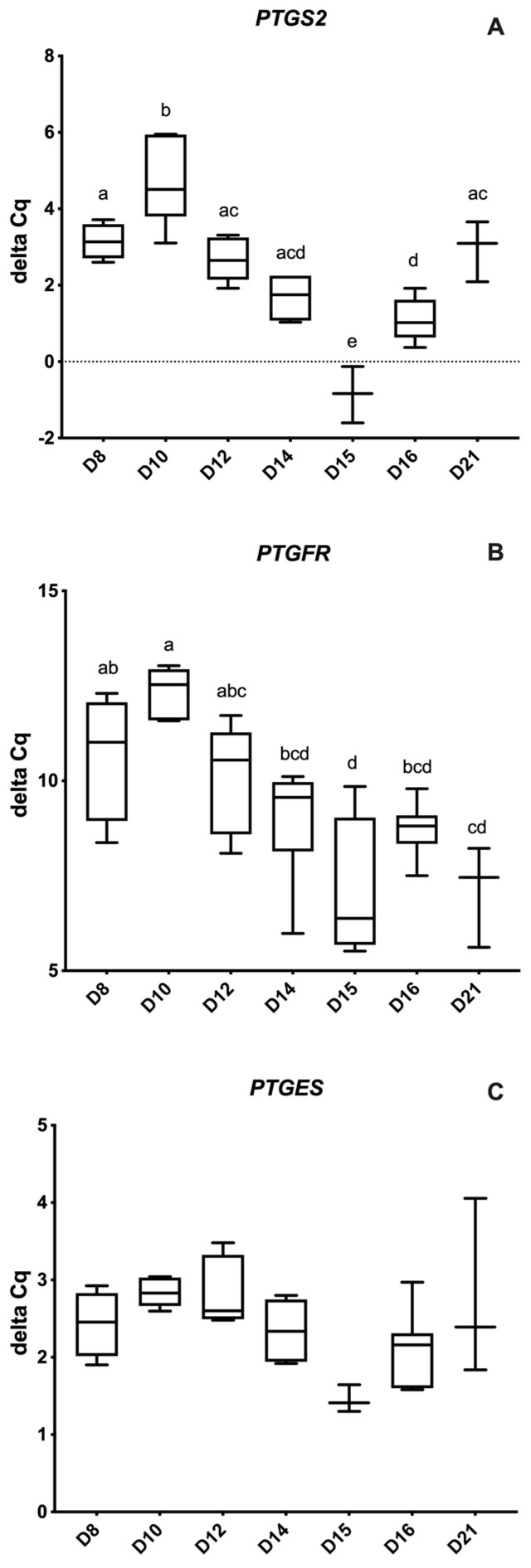
Relative abundance (delta Ct) of prostaglandin-related genes by embryonic age (Days 8 to 21 post-ovulation); (**A**) Prostaglandin-Endoperoxide Synthase 2 (*PTGS2*), (**B**) Prostaglandin F Receptor (*PTGFR*) and (**C**) Prostaglandin E Synthase (*PTGES*). Differences between days (*p* < 0.05) are indicated by different superscripted letters.

In relation to prostaglandin genes, PTGS2 and PTGFR had significantly lower and higher expression on D10 and D15, respectively (*p* < 0.001). Figure 4A–C demonstrates relative expression of PTGS2, PTGFR and PTGES genes among the different embryonic/conceptus stages.

#### 3.1.4. Glucose Transporter Genes

**Figure 5:**
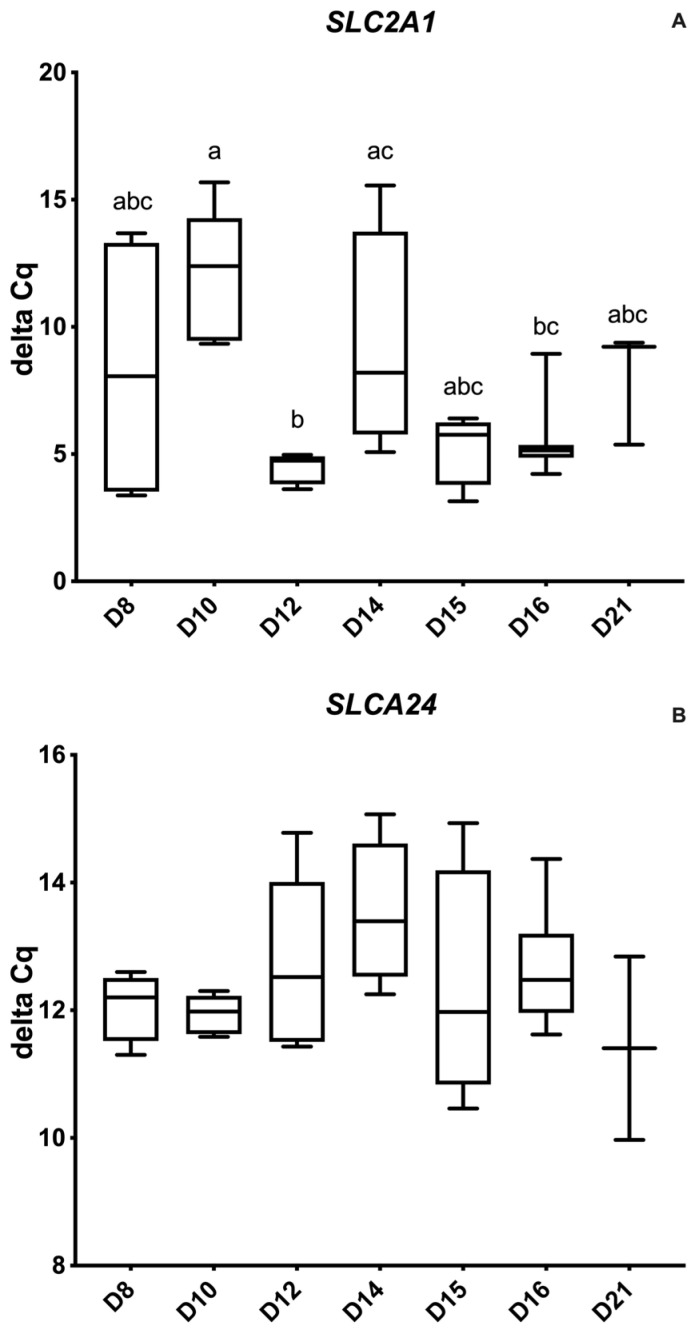
Relative abundance (delta Ct) of glucose transporter genes by embryonic age (Days 8 to 21 post- ovulation); (**A**) Glucose transporter protein type 1 (GLUT1) (SCL2A1) and (**B**) Glucose transporter protein type 4 (GLUT4) (SCL2A4). Delta Ct concentrations have an inverse relationship to mRNA abundance. Differences between days (*p* < 0.05) are indicated by different superscripted letters.

The glucose transporter SLC2A1 had much higher relative expression on D12 compared to D10 and D14, and D16 compared to D10 (*p* = 0.002). The lowest relative expression was on D8, D10 and D14 and higher abundances on D12, D15, D16 and D21 (Figure 5A,B). Furthermore, SLC2A1 was positively correlated to PTGS2 on D14 and 16 (r = 0.81) (*p* < 0.05) and to PTGES (r = 0.85) (*p* < 0.02). Day 14 conceptus SLC2A4 expression was positively correlated to PTGFR (r = 0.98) (*p* < 0.02).

#### 3.1.5. Gene Correlations

A correlation between the genes by embryonic/conceptus days was performed, and significant correlations are shown in Table 3. PGR was strongly negatively correlated (r = −0.99, *p* < 0.01) with ESR1 and positively correlated (r = 0.89, *p* = 0.04) with PTGS2 in D10 conceptus (Table 3). ESR1 and PGR were positively (r = 0.99, *p* = 0.0003) and negatively (r =−0.94, *p* = 0.02) correlated with OXTR on D10, respectively. Furthermore, ESR1 was positively correlated with OXT (r = 0.98, *p* = 0.03) on D12. OXT was negatively associated with PTGES on D10 (r = 0.99, *p* = 0.001) and D21 (r = 0.99, *p* = 0.02), but positively associated with PTGFR (r = 0.99, *p* = 0.05) on D21.

Regarding LNPEP, LNPEP was positively associated with PTGFR on D12 (r = 0.97, *p* = 0.006), D14 (r = 0.96, *p* = 0.003) and D15 (r = 0.99, *p* = 0.003). In addition, there was a positive association of LNPEP with SLC2A4 and with PTGES on D15 (r = 0.99, *p* = 0.01; r = 0.97, *p* = 0.03, respectively). OXTR was also positively correlated with PTGES on D15 (r = 0.99, *p* = 0.01).

The glucose transporters were positively correlated with prostaglandin-related genes. Briefly, SLC2A1 was positively correlated to PTGS2 on D14 and D16 (r = 0.8, *p* < 0.05), and to PTGES on D16 (r = 0.85, *p* < 0.02). The SLC2A4 was positively correlated to PTGFR (r = 0.98, *p* < 0.02) and PTGES (r = 0.88, *p* < 0.02) on days D15 and D16, respectively.

### 3.2. Western Blot

**Figure 6:**
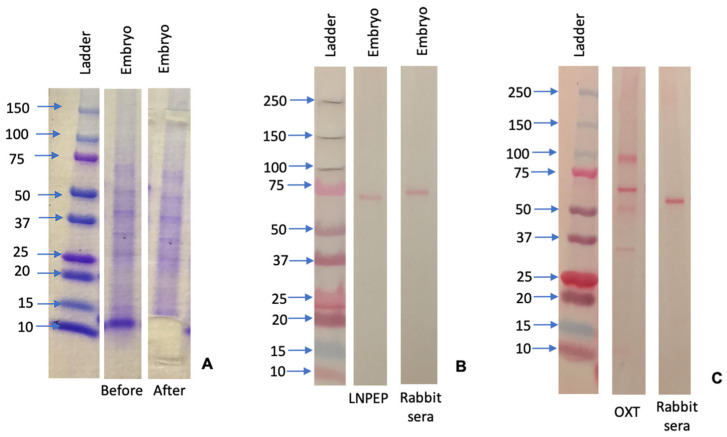
Coomassie blue gel demonstrating before and after band excision (**A**), and western blot gel demonstrating absence of leucyl-cystinyl aminopeptidase (LNPEP) (**B**) and oxytocin (OXT) with a parallel isotope negative control of rabbit sera (**C**).

A western blot of the homogenized conceptus tissue was performed, and no bands corresponding to the expected molecular size of LNPEP and OXT were identified. Figure 6A–C demonstrates the Coomassie blue gel with bands excised and the western blot with the negative-control rabbit sera gel.

### 3.3. Liquid Chromatography Tandem Mass-Spectrometry (LC-MS/MS)

The area of the polyacrylamide gel corresponding to the molecular weight of LNPEP and OXT was excised, and a proteomic analysis was performed. Using LC-MS/MS, 760 proteins were identified with at least two unique peptides. Leucyl and cystinyl aminopeptidase isoform X2 [*Equus caballus*] (LNPEP), accession number: 194216917 was identified with MW of 117.3KDa, but OXT was not identified with this technique (Supplementary Table S1).

### 3.4. Immunohistochemistry for LNPEP and OXT

**Figure 7:**
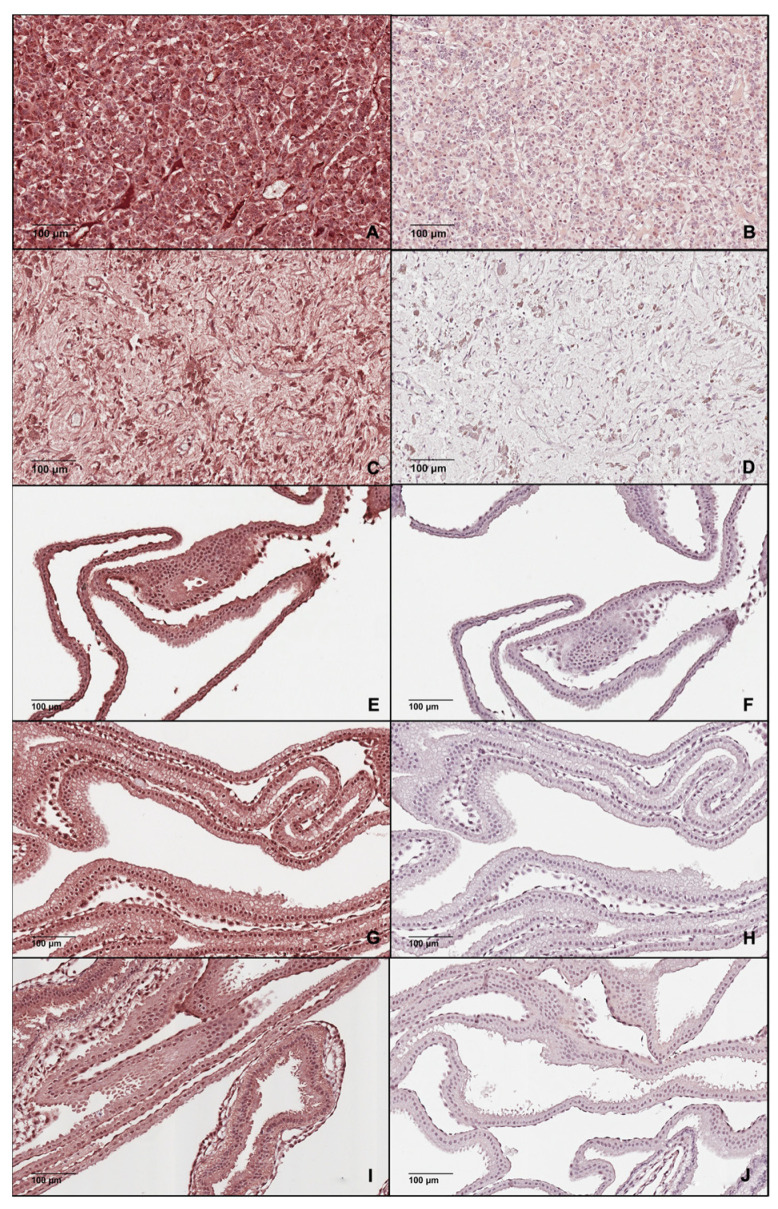
Photomicrograph of the equine adenohypophysis (**A**) and neurohypophysis (**C**) as the positive control tissues with intense staining for the Leucyl and cystinyl aminopeptidase (LNPEP) and its corresponding negative epitope control ((**B**,**D**), respectively) and the equine embryonic membranes at Day 15 (**E**,**F**), Day 16 (**G**,**H**) and Day 18 (**I**,**J**) post-ovulation demonstrating immunostaining for LNPEP of the trophectoderm and endoderm (**E**,**G**,**I**) and the corresponding negative epitope control (**F**,**H**,**J**).

**Figure 8:**
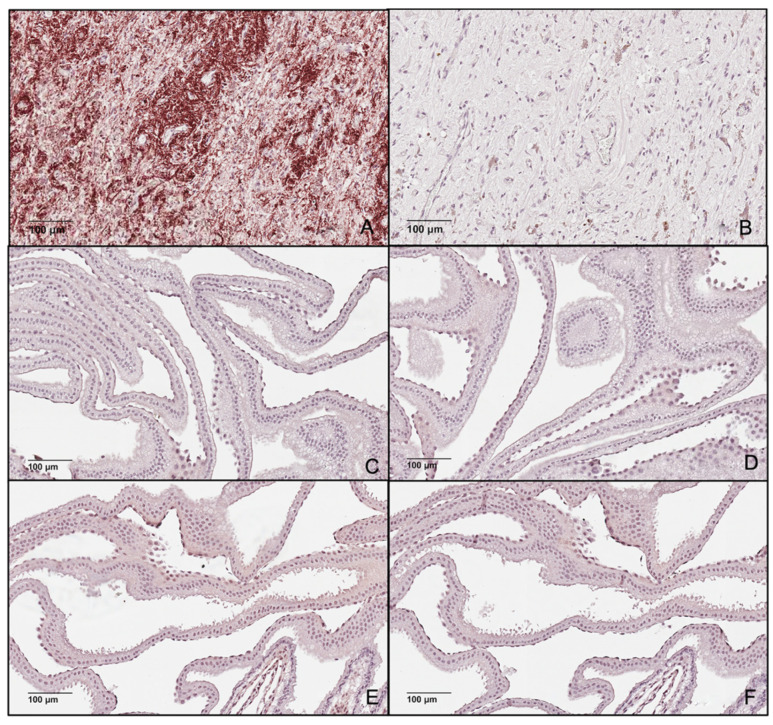
Photomicrograph of equine neurohypophysis (**A**) with positive staining for oxytocin with its corresponding negative epitope control (**B**) and the equine embryonic membranes at Day 16 and Day 18 post-ovulation demonstrating a lack of immunostaining for oxytocin 20× (**C**,**E**), and the corresponding negative epitope control (**D**,**F**).

Immunohistochemistry of equine Days 15, 16 and 18 trophoblast tissue was performed for LNPEP and D16 and D18 for OXT. Substitution of the primary antibody (negative controls) resulted in no immunostaining (Figure 7B,D,F,H,J and Figure 8B,D,F), with the exception of the cells of the endoderm, so this was considered nonspecific/background staining. The positive tissue control for LNPEP had intense staining of intravascular fluid, endothelium and cytoplasm of interstitial cells of the adenohypophysis and cytoplasm of cells and presumptive axons of the neurohypophysis (Figure 7A,C). There was intense staining of the nucleus and cytoplasm in trophectoderm and endoderm (Figure 7E,G,I).

The positive tissue control for oxytocin, the posterior pituitary, had positive cytoplasmatic-axonal immunostaining particularly around blood vessels in the neurohypophysis (Figure 8A). No immunostaining was identified for OXT in trophoblast (Figure 8C,E).

## 4. Discussion

We identified *LNPEP* in the equine embryo/conceptus using qPCR, and the protein using LC-MS/MS and immunohistochemistry. *LNPEP* expression was increased at D15, compared to the D10 conceptus, which is near the time of cessation of embryonic movement. There was strong immunostaining of the trophoblast for LNPEP protein that was both nuclear and cytoplasmic. LNPEP is reportedly a cytoplasmatic protein, however perinuclear immunostaining has been reported for the insulin-regulated aminopeptidase (IRAP), which is a mouse homologous peptidase to the human LNPEP [46], and nuclear immunostaining may be nonspecific. As embryo technologies such as gene knockout are not available for horses, the role of trophoblastic LNPEP is difficult to test directly in vivo. *LNPEP* was positively associated with conceptus *PTGFR* on D12, D14, D15, and *PTGES* on D15 along with *SLCA2A4* on D15. LNPEP has been reported to be present in reproductive tissues in sheep [47], and may regulate the availability of oxytocin to bind to *OXTR.* A decreased availability of systemic or uterine luminal oxytocin from the actions of conceptus and/or endometrial LNPEP [47] may attenuate oxytocin induced amplification of endometrial prostaglandin secretion [20] and aid in the prevention of luteolysis [48]. In Ishikawa cells, progesterone induced the secretion of LNPEP and production of PGE2 was increased slightly by oxytocin, but PGE2 was increased significantly by pretreatment with progesterone [49]. There are possible local autocrine or paracrine pathways that may involve regulation of oxytocin by LNPEP.

Oxytocin/neurophysin I has been reported to be expressed in other tissues, such as ruminant luteal tissue and equine endometrium, where it was immunolocalized to the superficial glands and luminal epithelium [32,50,51]. We detected *OXT* in the conceptus, but we did not detect oxytocin protein in the D8–D16 embryo/conceptus using various methodological approaches, including proteomics combined with LC-MS/MS and IHC. The IHC utilized a polyclonal rabbit anti-oxytocin antibody raised against synthetic oxytocin bound to thyroglobulin that we anticipated would recognize oxytocin and oxytocin/neurophysin I. Figure 8A, which is our positive control tissue, equine neurohypophysis, shows immunostaining of the neurohypophysis which stores both neurophysin bound oxytocin and free oxytocin [52]. The methodological approaches we used such as IHC and LC-MS/MS are able to detect very low levels of OXT. Protease inhibitors were used in sample processing, but we cannot exclude that there may have been rapid degradation of oxytocin by LNPEP or other proteases; however, we feel this is unlikely. We did not evaluate other conceptus membranes besides the trophoblast in this study. In other species, authors reported that *OXT* is not expressed in the trophoblast of: sheep D13–D23, cattle D16 and D30 and pigs D16, D18 [53,54]. Embryonic rat telencephalon has no *OXT* from D12–20 of development [55] and the D14–21 placenta had declining levels of *OXT*; however, only a consistent low level of oxytocin peptide was identified, which was immunolocalized to the trophoblast, cytotrophoblast and Giant cells [56].

Oxytocin was identified in the yolk sac fluid from 13–31 days and uterine flush fluid from cycling, diestrus and pregnant mares, with pregnant mares having the lowest amount of protein detected using a radioimmunoassay. The origin(s) of this immunoreactive oxytocin remains to be confirmed [10,31,51]. In embryonic rats, very low levels of immunoreactive oxytocin were identified using IHC and immunoassay of brain homogenates, but was not detected in the rat fetus in any quantity until the pups were postpartum D2. The developing brain is not a likely source of oxytocin [57,58].

Oxytocin exerts its actions through receptors, and *OXTR* was present in all embryos/conceptuses, similar to the findings of Budik et al., 2012 [26]. The conceptus *OXTR* on D10 is significantly associated (*p* = 0.0003) with *ESR1* abundance. We report decreased embryonic *OXTR* abundance on D12 and D14 (Figure 2). Budik and co-authors (2012) demonstrated that equine embryos (D10–D16) express an increased abundance of *OXTR* on D14 and D16 and, like our findings, report a lower abundance of *OXTR* in the D12 conceptus. They also reported stronger immunostaining in the D12 conceptus, indicating a divergence in *OXTR* abundance and OXTR protein. These events were followed by a subsequent decrease in immunostaining at D14 [26]. In our study, the highest relative abundance of conceptus *OXTR* was found on D21, after uterine transport of the conceptus had ceased. Binding of oxytocin to embryonic/conceptus OXTR would be predicted to augment PGE secretion, as *OXTR* was correlated to *PTGES* on D15. Recently, Budik and coworkers (2021) reported that trophoblast cells treated with oxytocin in an in vitro challenge had no difference in major prostaglandin gene abundance compared to non-treated controls, and this may be due to the presence of LNPEP [7].

Our study demonstrated *ESR1* expression in all the equine embryo/conceptus stages examined (D8–D21), with an increase on D12 and even higher expression at D21, consistent with the findings of Rambags and co-authors [4]. Estrogen from the conceptus was expected to bind to *ESR1* and further increase *ESR1* expression [59]. Conceptus estrogen secretion and *ESR1* expression have been reported to be related to higher levels of *OXTR* and prostaglandin secretion [14,26]. In this study, conceptus *ESR1* was correlated with *OXTR* on D10. The conceptus may engage paracrine mechanisms to regulate steroid receptors, as pregnant mares have a decreased endometrial expression of *ESR1* on D13.5, 14 and 15 compared to nonpregnant mares [60,61,62]. Similar to the conceptus *ESR1* pattern of expression, conceptus *PGR* expression was higher on D12 and D21; however, this was reversed on D10, where *ESR1* had lower expression and *PGR* higher expression, which then decreased by D14 [4]. Changes in *ESR1* suggest a role for conceptus estrogen potentially acting in an autocrine and paracrine matter with *LNPEP* to regulate both conceptus and endometrial factors such as prostaglandin and OXTR.

Equine conceptus prostaglandin secretion is suggested to mediate uterine contraction and therefore may play a role in propulsion of the conceptus [63]. Stout and Allen demonstrated that the equine conceptus yolk sac fluid has high concentrations of PGF2α and PGE2, with PGE2 levels being two to five times higher than PGF2α [14]. In the same study, the authors evaluated the concentration of PGF2α in the uterine flush fluid of pregnant and nonpregnant mares. They found that pregnant mares have negligible amounts of PGF2α during the expected time of luteolysis (D14–16), but PGF2α can be found in higher concentrations after D18 [14]. Conceptus expression of *PTGS2* and *PTGFR* started to increase around D12 and achieved high expression at D15, then decreased by D21, which is in agreement with Budik and co-authors [7]. The higher expression of *PTGS2* and *PTGFR* at D15 may be a preparatory event for conceptus fixation by causing maximal local endometrial vascular perfusion to ensure conceptus development will take place [64], or may be important in changing the PGE/PGF ratio, which may be of significance in supporting luteal function. The relative abundance of *PTGES* was not affected by embryonic/conceptus age, which is also in agreement with Budik et al. [7]. The contribution and paracrine effects of conceptus prostaglandin secretion relative to endometrial production remains to be elucidated [9]. Budik and co-authors have identified expression of carbonyl reductase 1 (*CBR1*) in the equine conceptus, but not *AKR1C1*, suggesting that the horse conceptus produces prostaglandin F2 alpha from PGE2 through CBR1 [7]. Further research on the prostaglandin transporters (PGT) and other related prostaglandin enzyme synthesis in the equine endometrium and conceptus is warranted. Prostaglandin transporters play an important role in the luteal maintenance in other species, such as pigs [65]. More studies on the role of LNPEP in regulating the effects of oxytocin on prostaglandin secretion by the conceptus are needed.

The glucose transporters and their role in fetal nutrition and growth are well studied in humans [66], and recently have been investigated in the equine endometrium and trophoblast [40]. Gibson and co-authors found an upregulation of *SLC2A1* in the endometrium of pregnant mares at D14 [40], but reported the downregulation of endometrial *SLC2A4.* They identified no difference in *SLC2A1* expression in the trophoblast at the gestational ages studied (D14, 21 and 28), and low expression for *SLC2A4.* Our study compared embryos/conceptuses of younger ages and we demonstrated higher *SLC2A1* expression at D12, with a decrease at D14 and subsequent increase again in later stage conceptuses (D15–21); no difference in *SLC2A4* was identified. It is unknown if the embryonic/conceptus *SLC2A1* is regulated by conceptus prostaglandins in the horse as it is in sheep, where endometrial *SLC2A1* is regulated by conceptus IFNτ and prostaglandin [38,39]. We demonstrated a positive correlation with conceptus prostaglandin synthases to *SLC2A1* on D14 and D16, and with *SLC2A4* and *PTGFR* on D15 and *PTGES* on D15 and D16. In addition, LNPEP is reported to be co-localized with the insulin responsive glucose transporters 4 (GLUT4) [36], and the GLUT4 vesicles move LNPEP to the surface of the cell where LNPEP may be released or membrane bound to regulate the activity of oxytocin [35,37]. Further research is also warranted on *LNPEP* and glucose transporters, to determine if LNPEP is co-localized with GLUT4 vesicles and if regulation of GLUT4 influences conceptus-maternal signaling.

Pituitary secretion of oxytocin is associated with large peaks of endometrial (PGF2α) [24] in mares, which would be predicted to induce myometrial contraction and movement of intrauterine objects such as embryos, marbles or similar devices. Superimposed on this pattern is embryo/conceptus-initiated myometrial activity related to the secretion PGF2α and PGE that produces independent movement [67,68]. The equine conceptus is transported to different locations in the uterus 10 to 20 times per day, with most movements reported at the time of MRP from D11 to 14 [67,69]. Budik et al., 2021 proposed that the embryo is anatomically polarized, with PGFα secreted at the embryonic pole producing a contractile effect and PGE2 secreted at the abembryonic pole producing a relaxant effect, generating a peristaltic wave that propels the embryo/conceptus [7]. As embryo/conceptus *PTGS2* and *PTGFR* increase from D12–15 and PTGFR remains high at D21, there must be regulatory event(s) that intercepts this process and results in embryo fixation. The increase in *PTGES* on D15 combined with *LNPEP* may be part of a conceptus signaling mechanism for fixation.

## 5. Conclusions

The conclusions of the study are limited by the small numbers of embryos/conceptuses, and further investigation of OXT and LNPEP protein in a larger number of embryos/conceptuses at different stages of early pregnancy is desirable. LNPEP was present in the equine embryo/conceptus and was immunolocalized to the trophoblast. Despite the presence of OXT, we did not identify oxytocin in the D15, 16 and 18 conceptus, which is consistent with the findings reported in other species. Changes in the temporal gene expression in the equine conceptus were prominent from D12 to D15, and the highest abundance for many genes was noted in the D21 conceptus. Higher abundance of conceptus LNPEP on D15 may be associated with the signaling mechanism associated with conceptus fixation, as the high levels of conceptus LNPEP is consistent with low levels of oxytocin and low levels of prostaglandin measured in uterine flush fluid of pregnant mares at the time of MRP [14,51].

## Figures and Tables

**Table 1:** Genes, accession number, forward (5′->3″) and Reverse (3->5) primers.

Symbol	Accession Number	Forward (5′ to 3′)	Reverse (5′ to 3′)
*ESR1*	NM_001081772.1	ACGATGCCACCAGACCATTT	CATGTGAACCAGCTCCCTGT
*PGR*	XM_001498494.4	GTCAGTGGACAGATGCTGTA	CGCCTTGATGAGCTCTCTAA
*OXTR*	XM_005600468.1	CATCGTGCTGGCCTTCATCGTGTG	GGTAGCCGGAGGAGCAGCAGAGGA
*OXT*		GATCCCTCCTGCGACCAC	CTATGGAGGGGACGAAGGGT
*LNPEP*	XM_001503684.4	CCTCAGCTCTCGGAGTAGGA	TGAAGCCGATCGTTGGTGAA
*PTGS2*	NM_001081775.2	GTGACATTGATGCCATGGAG	GAATGGTGCCCCAAGTTCTA
*PTGFR*	NM_001081806.1	CATAGGGCAGGTAGTTCTGCT	GACCCCAGTTTTTGAAGGCAA
*PTGES*	NM_001081935.1	CCACCCCCTAGCCTCGCGAT	GGCGAAAGCCTTCTTCCTCAGCC
*SLC2A4*	NM_001081866.2	TTCCTCATTGGCGCCTACTC	AATGACGATGGCCAGTTGGT
*SLC2A1*	NM_001163971.1	TCGCAGTCGGAGTCTCAGGA	GTTGTAGCCATACTGCAGGGA
*GAPDH*	NM_001163856.1	TTGTCAAGCTCATTTCCTGGTATG	GTTAGGGGGTCAAGTTGGGAC

Estrogen receptor 1 (*ESR1*), Progesterone receptor (*PGR*), Oxytocin receptor (*OXTR*), Oxytocin/neurophysin I prepropeptide (*OXT*), Leucyl and cystinyl aminopeptidase (*LNPEP)*, Prostaglandin-endoperoxide synthase 2 (*PTGS2*), Prostaglandin F receptor (*PTGFR*), Prostaglandin E synthase (*PTGES*), Solute carrier family 2 member 4 (*SLC2A4*), Solute carrier family member 2A1 (*SLC2A1*) and Glyceraldehyde-3-phosphate dehydrogenase (GAPDH).

**Table 2:** Comparisons by day post-ovulation of selected equine embryonic genes at *p* < 0.05.

Gene ^a^	Day	by Day	Difference	SED ^b^	Lower CL ^c^	Upper CL ^c^	*p*-Value
*ESR1*	8	21	6.64	1.49	1.86	11.41	0.003
	10	21	9.10	1.37	4.71	13.48	0.0001
	10	16	3.77	0.96	0.71	6.84	0.01
	10	12	3.40	1.03	0.09	6.71	0.04
	12	21	5.70	1.37	1.31	10.08	0.005
	14	21	8.40	1.34	4.13	12.68	0.0001
	14	16	3.08	0.91	0.17	5.99	0.03
	15	21	6.28	1.42	1.75	10.82	0.003
	16	21	5.32	1.31	1.12	9.52	0.007
*PGR*	15	21	3.42	1.04	0.12	6.72	0.04
	15	12	2.64	0.80	0.08	5.20	0.04
	16	21	3.26	0.96	0.21	6.32	0.03
	16	12	2.48	0.70	0.25	4.72	0.02
*OXTR*	10	21	5.42	1.45	0.73	10.10	0.02
	14	21	6.03	1.41	1.46	10.61	0.005
*OXT*	12	21	3.11	0.84	0.44	5.78	0.01
	14	21	3.17	0.81	0.59	5.76	0.01
*LNPEP*	10	15	1.77	0.54	0.03	3.50	0.04
*PTGS2*	8	15	4.01	0.55	2.26	5.75	0.0001
	8	16	2.06	0.45	0.63	3.49	0.001
	8	14	1.46	0.46	−0.01	2.93	0.05
	10	15	5.66	0.52	3.99	7.32	0.0001
	10	16	3.71	0.42	2.37	5.04	0.0001
	10	14	3.11	0.43	1.73	4.49	0.0001
	10	12	2.11	0.45	0.67	3.55	0.001
	10	21	1.85	0.52	0.18	3.51	0.02
	10	8	1.65	0.48	0.12	3.18	0.03
	12	15	3.55	0.52	1.88	5.21	0.0001
	12	16	1.60	0.42	0.26	2.93	0.01
	14	15	2.55	0.51	0.93	4.16	0.001
	16	15	1.95	0.49	0.38	3.52	0.01
	21	15	3.81	0.58	1.95	5.67	0.0001
	21	16	1.86	0.49	0.29	3.43	0.01
*PTGFR*	8	15	3.64	0.95	0.63	6.65	0.01
	8	21	3.58	1.02	0.33	6.83	0.02
	10	15	5.29	0.90	2.43	8.14	0.0001
	10	21	5.22	0.98	2.11	8.33	0.0002
	10	16	3.61	0.78	1.11	6.10	0.001
	10	14	3.32	0.81	0.74	5.90	0.01
	12	15	3.02	0.90	0.17	5.88	0.03
*SLC2A1*	10	12	22.40	4.84	7.01	37.79	0.001
	14	12	16.40	4.63	1.67	31.13	0.02
	10	16	16.00	4.48	1.75	30.25	0.02

^a^ Estrogen receptor 1 (*ESR1*), Progesterone receptor (*PGR*), Oxytocin receptor (*OXTR*), Oxytocin/neurophysin I prepropeptide (*OXT*), and Leucyl and cystinyl aminopeptidase (*LNPEP*), Prostaglandin-endoperoxide synthase 2 (*PTGS2*), Prostaglandin F receptor (*PTGFR*), Prostaglandin E synthase (*PTGES*), Solute carrier family 2 member 4 (*SLC2A4*) and Solute carrier family member 2A1 (*SLC2A1*); ^b^ standard error of the difference (SED); ^c^ confidence limit (CL).

**Table 3:** Correlation between equine embryonic age in days post-ovulation and gene expression of selected embryonic genes.

Embryonic Age (D) ^a^	Gene Variable	by Gene Variable	Correlation	Lower 95% CI ^b^	Upper 95% CI ^b^	*p*-Value
8	*PGR*	*SLC2A4*	−0.97	−0.99	−0.07	0.03
	*OXT*	*PTGES*	−0.97	−0.99	−0.10	0.03
10	*PGR*	*ESR1*	−0.97	−0.99	−0.52	0.01
	*PGR*	*PTGS2*	0.89	0.06	0.99	0.04
	*OXTR*	*ESR1*	0.99	0.94	0.99	0.0003
	*OXTR*	*PGR*	−0.94	−0.99	−0.30	0.02
	*OXT*	*PTGES*	−0.99	−0.99	−0.88	0.001
12	*OXT*	*ESR1*	0.98	0.73	0.99	0.003
	*LNPEP*	*PTGFR*	0.97	0.61	0.99	0.006
14	*LNPEP*	*PTGFR*	0.96	0.64	0.99	0.003
	*SLC2A1*	*PTGS2*	0.81	0.01	0.98	0.05
	*SLC2A1*	*LNPEP*	0.84	0.09	0.98	0.04
15	*PTGFR*	*SLC2A4*	0.98	0.33	0.99	0.02
	*PTGES*	*PTGFR*	0.98	0.37	0.99	0.02
	*OXTR*	*PTGES*	0.99	−1		0.01
	*LNPEP*	*SLC2A4*	0.99	0.55	0.99	0.01
	*LNPEP*	*PTGFR*	0.99	0.84	1	0.003
	*LNPEP*	*PTGES*	0.97	0.13	0.99	0.03
16	*PTGES*	*SLC2A4*	0.88	0.23	0.99	0.02
	*PTGES*	*PTGS2*	0.93	0.59	0.99	0.002
	*SLC2A1*	*SLC2A4*	0.85	0.13	0.98	0.03
	*SLC2A1*	*PTGS2*	0.80	0.12	0.97	0.03
	*SLC2A1*	*PTGES*	0.85	0.27	0.98	0.02
21	*PTGES*	*PTGFR*	−0.99	−1		0.03
	*OXT*	*PTGFR*	0.99	−1		0.05
	*OXT*	*PTGES*	−0.99	−1		0.02

Estrogen receptor 1 (ESR1), Progesterone receptor (PGR), Oxytocin receptor (OXTR), Oxytocin/neurophysin I prepropeptide (OXT), Leucyl and cystinyl aminopeptidase (LNPEP), Prostaglandin-endoperoxide synthase 2 (PTGS2), Prostaglandin F receptor (PTGFR), Prostaglandin E synthase (PTGES), Solute carrier family 2 member 4 (SLC2A4) and Solute carrier family member 2A1 (SLC2A1). ^a^ Day; ^b^ confidence interval.

## Data Availability

Data can be made available upon request.

## Supplementary Materials

The following supporting information can be downloaded at: https://www.mdpi.com/article/10.3390/ani12070799/s1, Table S1: List of selected proteins detected by LC-MS/MS excised from the polyacrylamide gel at 10–15 KDa and 120–150 KDa. Note: leucyl and cystinyl aminopeptidase (LNPEP) was identified (indicated by the black border) with the expected molecular weight (MW) of 117.3, but oxytocin/neurophysin-1 was not detected.
